# Default Mode Network Alterations Induced by Childhood Trauma Correlate With Emotional Function and SLC6A4 Expression

**DOI:** 10.3389/fpsyt.2021.760411

**Published:** 2022-01-27

**Authors:** Tian Tian, Jia Li, Guiling Zhang, Jian Wang, Dong Liu, Changhua Wan, Jicheng Fang, Di Wu, Yiran Zhou, Yuanyuan Qin, Wenzhen Zhu

**Affiliations:** ^1^Department of Radiology, Tongji Hospital, Tongji Medical College, Huazhong University of Science and Technology, Wuhan, China; ^2^Tongji Hospital, Tongji Medical College, Huazhong University of Science and Technology, Wuhan, China

**Keywords:** anxiety, harm avoidance, default mode network, childhood trauma, gene expression, functional connectivity

## Abstract

As one of the most studied resting-state functional networks, default mode network (DMN) is related to pathogenesis in neuropsychiatry. However, it is unclear whether changed DMN connectivity is transformed into vulnerability to psychopathology in adults who experienced childhood trauma, and what is the underlying genetic basis. Exploring the effect of DMN on environment-behavior pathway and the related genetic modulation mechanisms could further a better understanding of psychiatric pathogenesis and early prevention strategy. Two hundred and sixteen young adults with varying levels of early trauma indexed by the Childhood Trauma Questionnaire (CTQ) were recruited from the community. Static and dynamic functional connectivity based on DMN seeds and independent component analysis based on whole-brain voxels were combined to explore DMN alterations related to the CTQ score. Relationships between CTQ score, DMN connectivity, and behavioral scores were confirmed by mediation effect analysis. Imaging-genomic correlations were further used to identify risk genes whose expression was associated with the DMN changes. Dysregulated DMN connectivity was found both in seed-level and voxel-level analyses. Moreover, the functional disruption in the left temporal pole, right parahippocampal gyrus, and frontoparietal connectivity mediated the effects of childhood trauma on emotional behavior. The serotonin transporter gene was identified and might suggest the biological underpinning of the relationship between childhood trauma, DMN, and emotion regulation. Changed DMN may be useful as biomarkers to provide a powerful supplement to psychological evaluation related to childhood trauma. Combined with gene expression profiles, our findings advance a more integrative understanding of DMN alterations induced by childhood trauma, and clarify its implications for psychiatric pathogenesis and early prevention strategies.

## Introduction

The brain's “default mode network” (DMN) is a popular and rapidly growing neuroscientific topic of mental health. Now regions of the DMN have been more specifically related to autobiographical memory retrieval, perception of the mental states of others, navigating social interactions, mentalizing, self-projection, planning for the future, and passive cognitive states (1–3). The variety of perspectives attributing specific functions to the DMN underscores its importance in the field of psychiatry (4, 5).

Childhood trauma, defined as perceived neglect or abuse in early environment, is reported to increase the vulnerability to psychopathology in adulthood (6). However, the mechanisms by which cognitive or emotional biases translate into vulnerability are unclear. As a gradually maturing architecture across childhood and adolescence (7), the DMN connectivity may be modified by childhood trauma experience (8). During the sensitive period of brain-development, early trauma experience can act as life stressor and produce a series of hormonal changes and physiological reactions, which may cause permanent modification to neural structure and function (9, 10), including DMN regions. The relationship between early trauma, disturbances in DMN regions, and psychopathology has been uniformly confirmed (11–14). Neuroimaging studies highlight the functional plasticity of DMN during the early sensitive or critical periods (8, 15), and support the model of dysregulated DMN connectivity in early life trauma and related negative affect states. However, there is a lack of research on how DMN connectivity mediates the impact of childhood trauma on mental distress severity. Further, the existence and nature of DMN dysfunction caused by childhood trauma experience are less clear in clinical studies if psychiatric conditions and the interaction between childhood trauma and psychiatric disorders are not controlled (11, 12, 16, 17). Therefore, preclinical studies are critical for understanding the relationship between DMN, childhood trauma, and risk of mental illness.

In this study, we recruited 216 young adults from the community, who completed Childhood Trauma Questionnaire (CTQ), behavioral scores, and resting-state functional magnetic resonance imaging (fMRI). Static and dynamic functional connectivity based on DMN seeds as well as independent component analysis based on whole-brain voxels were combined to explore a more integrative understanding of DMN functional alterations, which were further analyzed in the environment-brain-behavior mediation model. Moreover, there have been dozens of reports of the important role of genes in predicting psychopathology related to childhood trauma (18, 19). A link between molecular function to macroscale brain organization has given rise to the new field of imaging transcriptomics (20). Based on mRNA gene expression profiles derived from the Allen Human Brain Database (ABA) (21, 22), we also identified risk genes whose expression was associated with the DMN changes. Our aims were exploring (a) whether functional connectivity of DMN obtained by different analytical techniques was associated with childhood trauma experience; (b) whether the DMN alterations mediated the environment-behavior pathway, leading to an increased risk for psychopathology; (c) how imaging-genomic correlations shed light on the biological underpinning of the relationship between the DMN changes, childhood trauma, and potentially psychopathologies.

## Materials and Methods

### Participants and Questionnaires

A total of 216 young adults were recruited from the community. Recruitment criteria included: (a) all participants had no history of genetic psychiatric or neurological illness, psychiatric treatment, or drug or alcohol abuse; (b) no contraindications for MRI examination; (c) Chinese Han populations; and (d) strongly right-handed. The human experiment was approved by the Ethical Committee of Tongji Hospital of Tongji Medical College of Huazhong University of Science and Technology. Written informed consent was obtained from each subject before the study.

Childhood trauma experiences were assessed by administering the Chinese version of the CTQ-Short Form (23, 24). Participants also completed California Verbal Learning Test-Second Edition, Beck Depression Inventory (BDI), Tridimensional Personality Questionnaire, and Spielberger's State-Trait Anxiety Inventory (STAI) to further characterize subjects.

### MRI Data

All scans were performed on a 3.0-Tesla MR system (Discovery MR750, General Electric, Milwaukee, WI, USA). Tight but comfortable foam padding was used to minimize head motion, and earplugs were used to reduce scanner noise. Resting-state fMRI data were obtained using Single-Shot Echo-Planar Imaging. To better coregister the fMRI data, sagittal 3D T1-weighted images were acquired using a brain volume sequence. Detailed parameters and data preprocessing procedure were the same as described in our previous study (25).

### Static and Dynamic Functional Connectivity Analysis

Region of interest (ROI)-ROI correlation analysis was carried out by the CONN (RRID:SCR_009550) connectivity toolbox (26) running on Matlab 2017a. We used the same seeds to define the DMN as in a previous study (7). The MNI coordinates of 13 cerebral seeds are shown in Supplementary Table S1. All ROIs were defined as a spherical region with a radius of 6 mm at the center of the obtained coordinates of a specific seed. Supplementary Figure S1 shows the distribution of each seed on a three-dimensional map.

**Figure 1:**
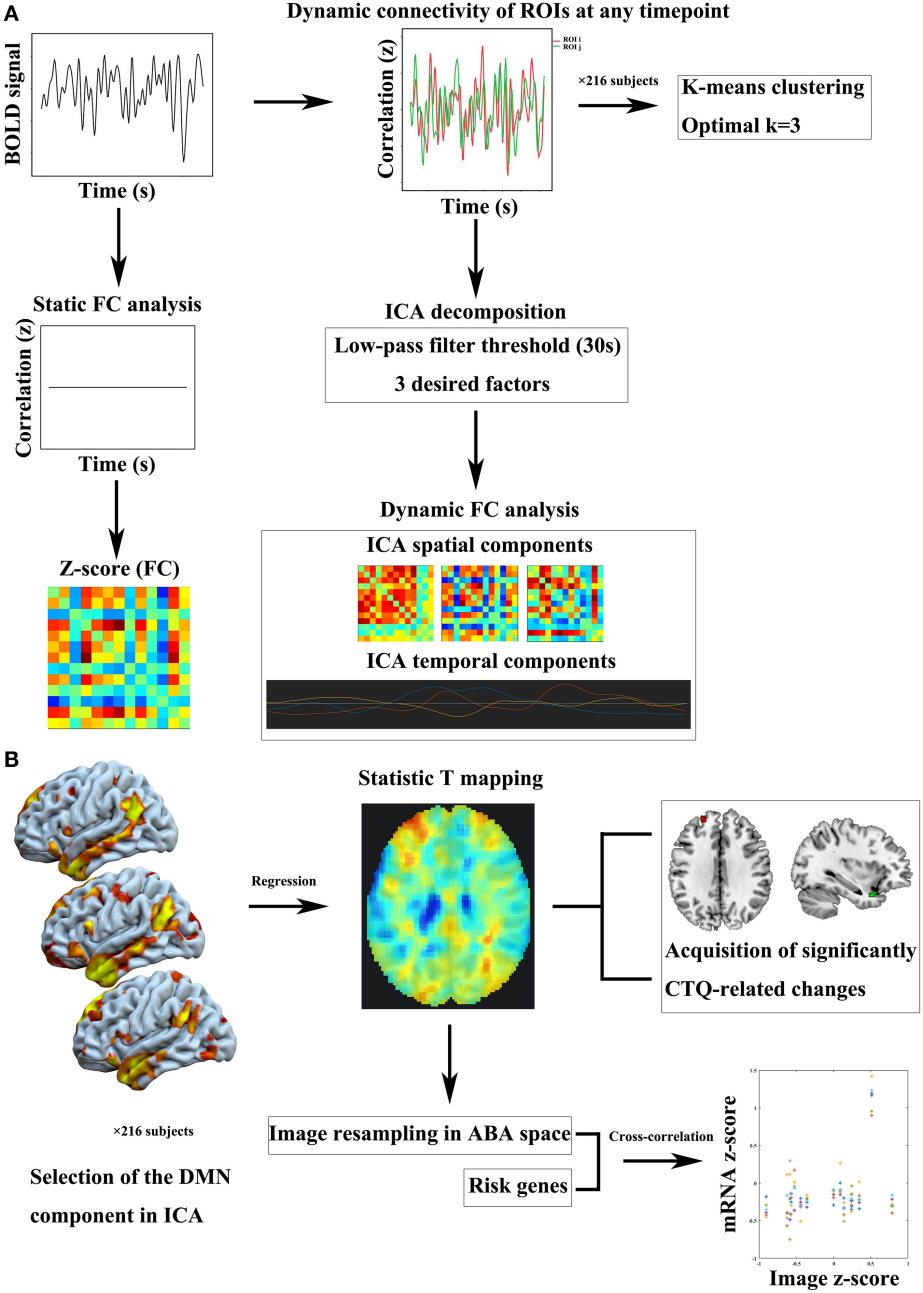
Flow charts of imaging analysis and imaging-genomic correlation analysis. **(A)** Static and dynamic FC processes. **(B)** Intra-DMN FC analysis based on ICA approach and imaging-genomic correlation analysis. BOLD, blood oxygen level-dependent; CTQ, Childhood Trauma Questionnaire; DMN, default mode network; FC, functional connectivity; ICA, independent component analysis; ROI, region of interest.

For static functional connectivity, the mean time series was extracted by averaging the fMRI time series of the entire acquisition throughout the voxels within the same region. The static functional connectivity was generated by averaging the blood oxygen level-dependent (BOLD) time series separately in the two ROIs and then computing the Pearson's correlation coefficient between the two averaged time series. Dynamic connectivity analyses performed in the CONN connectivity toolbox explored dynamic properties (temporal modulation) of the ROI-ROI connectivity matrix, and identified a number of circuits of similarly-modulated connections. We performed an ICA of the strength of connectivity between each pair of ROIs at any given timepoint, returning a number of independent components that best characterized the observed functional connectivity modulation across time. We adopted k-means algorithm to define the number of desired components. *K*-means clustering applied Manhattan distance and was repeated 500 times to regroup similarly-modulated connections. The optimal number of clusters was estimated using the elbow criterion, calculating the ratio of within-cluster distance to between-cluster distance. Finally, three desired components were determined. Previous studies have shown that fluctuations in dynamic functional connections can be successfully captured in the range of 30–60 s window-length (27, 28). In our study, a 30-s low-pass filter threshold (similar to window-length in sliding-window approaches) was entered in order to focus the estimated temporal modulation factors on the desired low-frequency range. A simple flow chart of this part is provided in Figure 1A.

Topological metrics were further analyzed. The static and dynamic ROI-ROI functional connectivity values were computed and thresholded at a fixed network-level *z*-score value (0.15) to define an undirected graph. Topological metrics of network characteristics included the global efficiency, local efficiency, betweenness centrality, cost, average path length, clustering coefficient, and degree.

### Intra-DMN Functional Connectivity Analysis of ICA Based on Whole-Brain Voxels

The primary process of this part is shown in Figure 1B. Calhoun's group-level ICA approach (29) was carried out by the CONN connectivity toolbox. Spatial match to template computed the correlation between each group-level spatial map and the CONN's default networks mask file, which was used to identify the DMN component. Then we performed correlation analysis between the CTQ and intra-DMN functional connectivity.

### Imaging-Genomic Correlation Analysis

During estimating correlations between the CTQ and DMN component, multiple linear regression generated a whole-brain statistic T mapping, which was further processed in MENGA software platform to investigate correlation patterns between the T mapping and mRNA gene expression profiles derived from the ABA (21). The image intensity data was normalized to *z*-scores and resampled in the genomic space independently for each donor of the ABA database. The list of genes included a large number of psychiatric risk genes identified in previous studies associated with childhood trauma (provided in Supplementary Table S2). For the region selection, we used the default region list corresponding to the simplified ABA coarse level of resolution in MENGA. For each region, a single mRNA value was hence calculated from the average of the samples within the region. For the image masking, our analysis came with the default mask derived from the FSL MNI ICBM152 template, limited to the left hemisphere, to benefit from the contribution of all donors. The cross-correlation analysis consisted of the weighted regression of mRNA and image data for each donor. The weights were defined as the ratio of the number of samples in each region over the variability of the image data in that region for each subject. The squared Pearson's correlation coefficient (*R*^2^) for each risk gene was reported in the summary statistics as mean and standard deviation across the different donors. A simple flow chart of this part is shown in Figure 1B. Next, functional annotation was conducted by a gene annotation and analysis resource named Metascape (https://metascape.org/gp/index.html, RRID:SCR_016620) (30) to explore the functional relevance of the identified genes.

### Statistical Analysis

The main effects of CTQ score on static and dynamic ROI-ROI connectivity were assessed by multiple linear regression model in the CONN toolbox, respectively. Sex, education level, and age were controlled in multiple regression analyses. Statistical significance was set at *p* < 0.05 false discovery rate (FDR) seed-level correction. In addition, the multiple linear regression model for graph-theory results was the same as above; statistical significance was set at *p* < 0.05 FDR correction.

For the whole-brain voxel-level analysis, firstly, each DMN component of each subject was entered into a random-effect one-sample *T*-test using a family-wise error correction (*p* < 0.05) in SPM12 (http://www.fil.ion.ucl.ac.uk/spm, RRID: SCR_007037) to generate a sample-specific spatial mask for further intra-network analysis. We investigated the correlation between the CTQ and intra-network functional connectivity using a multiple linear regression model. Sex, education level, and age were controlled. For each cluster on the basis of the whole-brain findings (uncorrected *p* < 0.01; cluster size > 5 voxels), a 10-mm box dimension centered at the peak location of the cluster was placed around each cluster, and the small-volume family-wise error correction (*p* < 0.05) was used to correct for multiple comparisons.

In the imaging-genomic correlation analysis, for the simplified coarse region list, the correlation is significant for a correlation coefficient *R*^2^ > 0.25 (*p* < 0.05, Bonferroni correction) recommended by the software developer.

Statistical analyses for the demographic and behavioral data were performed using Statistical Package for the Social Sciences version 18.0 (SPSS, Chicago, IL, USA). Considering the influence of gender, two-sample *t*-test were applied to test gender differences in behavioral scores. We used a linear regression model with age, education level, and sex as covariates to test the effect of CTQ score on behavioral data. Furthermore, in order to determine the relationship between the DMN, CTQ, and behavioral data, we used the SPSS macro to perform the mediation effect analysis (http://www.processmacro.org/index.html) based on a three-variable mediation model. We adopted the bootstrapping method to assess the significance of the mediation effect. According to the distribution of the indirect effect after 5,000 bias-corrected bootstrapping, we calculated 95% confidence intervals for the effect. If zero did not fall between the resulting 95% confidence interval, we concluded that there was a significant mediation effect.

## Results

### Demographic Characteristics and Behavioral Results

See Table 1 for sociodemographic and behavioral characteristics of the sample. We found that the CTQ score was positively correlated with the state anxiety score (β = 0.356, *p* < 0.001, *t* = 5.572), trait anxiety score (β = 0.346, *p* < 0.001, *t* = 5.523), BDI score (β = 0.289, *p* < 0.001, *t* = 4.419), and harm avoidance (HA) score (β = 0.204, *p* = 0.002, *t* = 3.118). There were no significant gender differences in behavioral scores (*p* > 0.05).

**Table 1:** Demographic and behavioral characteristics of the sample.

**Demographics**	**Mean (SD)**	**Range**
Age (years)	24.1 (1.9)	20–30
Gender (female/male)	158/58	NA
Education level (years)	17.7 (1.5)	13–22
CTQ	30.4 (5.2)	25–49
BDI	4.7 (4.9)	0–22
**STAI**		
State anxiety	32.6 (8.1)	20–57
Trait anxiety	36.5 (7.7)	21–63
**TPQ**		
Novelty seeking	13.9 (4.4)	4–29
Harm avoiding	15.2 (5.6)	2–30
Reward depending	19.1 (3.4)	10–27
**Verbal memory**		
Learning	59.4 (8.6)	23–79
Recall	13.4 (2.2)	5–16
Recognition	15.5 (0.9)	11–16
List A-1	7.6 (2.0)	3–15
List B	7.3 (2.3)	3–14
False positive errors	1.1 (1.9)	0–10

*BDI, Beck Depression Inventory; CTQ, Childhood Trauma Questionnaire; NA, not applicable; SD, standard deviation; STAI, State-Trait Anxiety Inventory; TPQ, Tridimensional Personality Questionnaire*.

### Static Functional Connectivity Between ROIs

**Figure 2:**
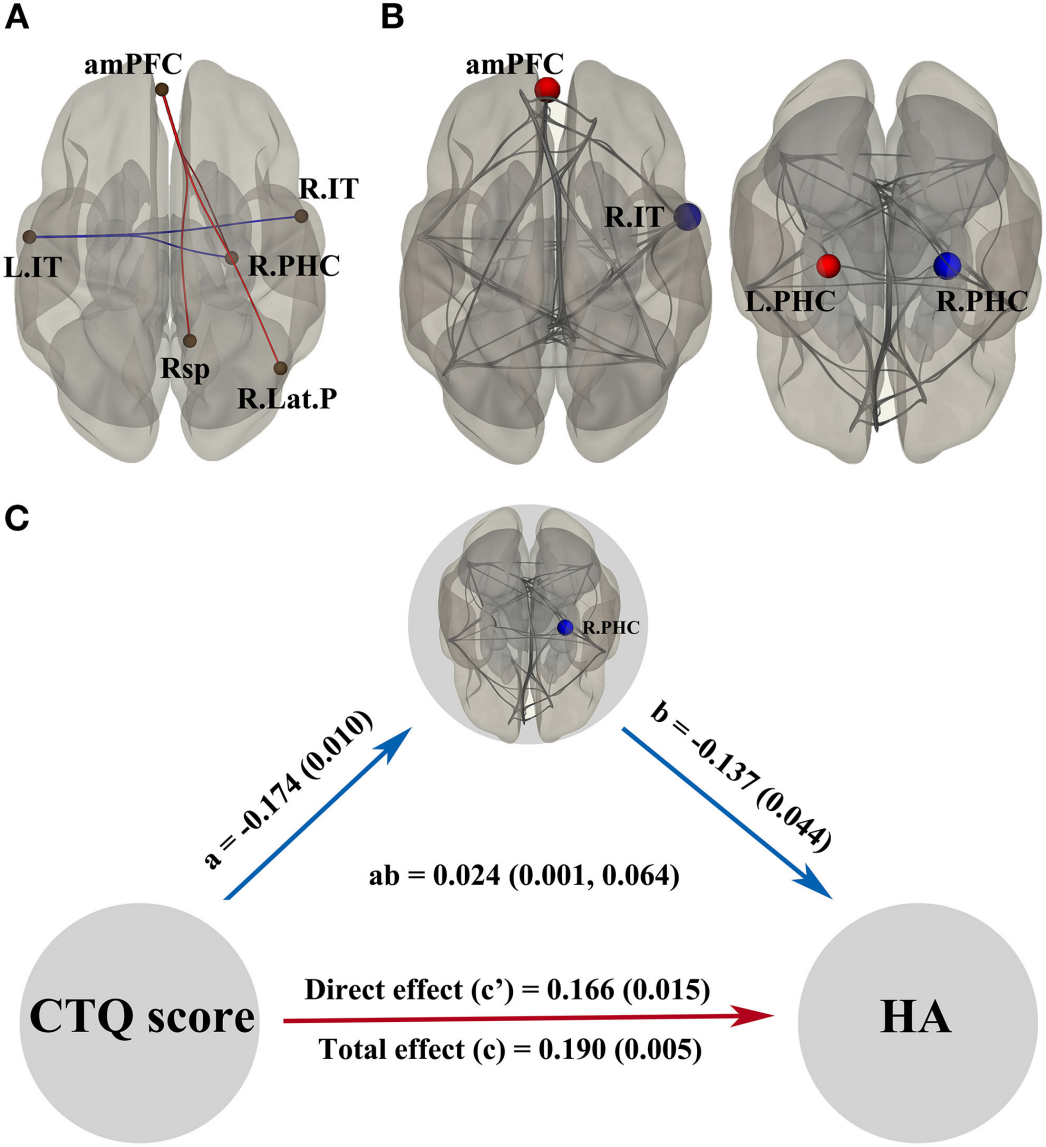
Static functional properties of DMN related to the CTQ score and significant mediation effects in the environment-brain-behavior pathway. In **(A,B)**, the red line/dot represents positive correlations with the CTQ score, while the blue line/dot represents negative correlations. The betweenness centrality in R.PHC was found as a significant mediator between the CTQ score and HA score **(C)**. Significant indirect effects were labeled with path coefficients and 95% confidence intervals, while the direct and total effects were labeled with path coefficients and *p*-values. There were significant positive effects from the CTQ score to HA score, a negative effect from the CTQ score to betweenness centrality in R.PHC, and a negative effect from the betweenness centrality in R.PHC to HA score. amPFC, anterior medial prefrontal cortex; CTQ, Childhood Trauma Questionnaire; DMN, default mode network; HA, harm avoidance; L.IT, left inferior temporal cortex; L.PHC, left parahippocampal gyrus. R.IT, right inferior temporal cortex; R.Lat.P, right lateral parietal cortex; R.PHC, right parahippocampal gyrus; Rsp, retrosplenial cortex.

Multiple linear regression analysis showed static functional properties of DMN related to the CTQ score in Figure 2A. Connections between anterior medial prefrontal cortex and right parahippocampal gyrus (*t* = 2.49), anterior medial prefrontal cortex and retrosplenial cortex (*t* = 2.55), anterior medial prefrontal cortex and right lateral parietal cortex (*t* = 2.30), left inferior temporal cortex and right inferior temporal cortex (*t* = −2.49), as well as left inferior temporal cortex and right parahippocampal gyrus (*t* = −2.71) were significantly correlated with the CTQ score, respectively. Figure 2B shows alterations of the DMN by topological metrics measurement. The CTQ score was significantly related to the betweenness centrality in right parahippocampal gyrus (*t* = −2.82), betweenness centrality in left parahippocampal gyrus (*t* = 2.45), cost in anterior medial prefrontal cortex (*t* = 3.15), degree in anterior medial prefrontal cortex (*t* = 3.15), efficiency in anterior medial prefrontal cortex (*t* = 2.44), and efficiency in right inferior temporal cortex (*t* = −2.60), respectively. In mediation analysis, only the betweenness centrality in right parahippocampus was found as a significant mediator between the CTQ score and HA score (Figure 2C).

### Dynamic Functional Connectivity Between ROIs

**Figure 3:**
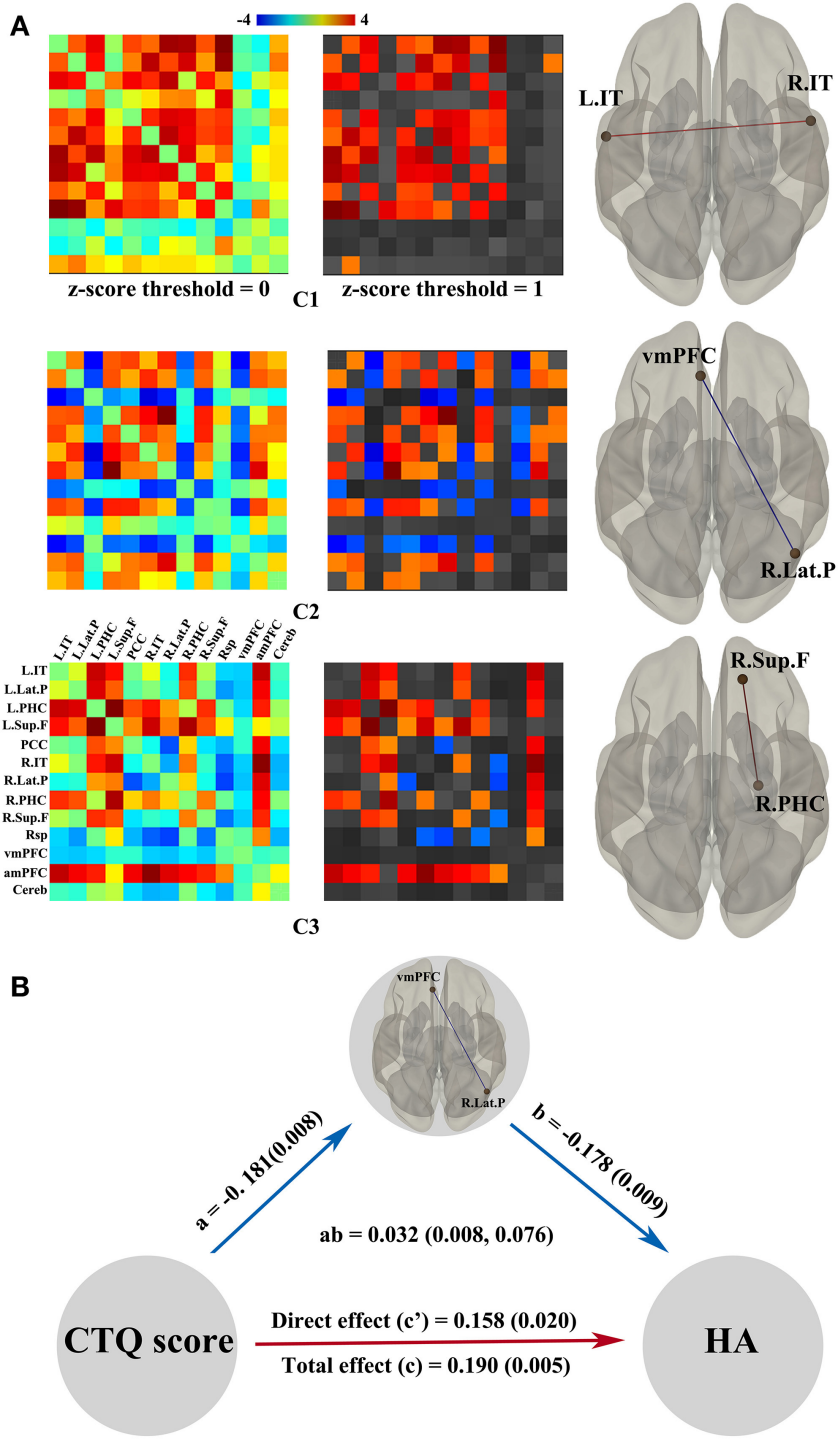
Dynamic functional properties of DMN related to the CTQ score and significant mediation effects in the environment-brain-behavior pathway. **(A)** shows three independent spatial components best characterizing the observed connectivity-modulation across time. Regression analyses showed connections that significantly correlated with the CTQ score in each component. The red line represents positive correlations while the blue line represents negative correlations. Only the vmPFC-R.Lat.P connectivity was found as a significant mediator between the CTQ score and HA score **(B)**. There were significant positive effects from the CTQ score to HA score, a negative effect from the CTQ score to vmPFC-R.Lat.P connectivity, and a negative effect from the vmPFC-R.Lat.P connectivity to HA score. CTQ, Childhood Trauma Questionnaire; DMN, default mode network; HA, harm avoidance; L.IT, left inferior temporal cortex; R.IT, right inferior temporal cortex; R.Lat.P, right lateral parietal cortex; R.PHC, right parahippocampal gyrus; R.Sup.F, right superior frontal cortex; vmPFC, ventral medial prefrontal cortex.

Figure 3A shows independent spatial components characterizing the observed connectivity-modulation across time. To better display the unique connection pattern of each component, we kept stronger connections within the DMN based on a higher *z*-score threshold (*z*-score threshold = 1). In component 1, all strong connections were positive and dispersive outside the medial prefrontal cortex; regression analysis showed the connectivity between left inferior temporal cortex and right inferior temporal cortex was significantly positively correlated with the CTQ score (*t* = 2.71). Component 2 exhibited distributed strong connections, both positive and negative; regression analysis showed the connectivity between ventral medial prefrontal cortex and right lateral parietal cortex was significantly negatively correlated with the CTQ score (*t* = −2.72). Component 3 showed a transitional connection pattern between component 1 and component 2; regression analysis found the functional connectivity between right superior frontal cortex and right parahippocampal gyrus was significantly positively correlated with the CTQ score (*t* = 3.28). For temporal properties associated with each spatial component, both temporal variability and frequency did not show meaningful correlations with the CTQ score. In addition, no significant correlation between the CTQ and topological metrics measurement was found in dynamic connectivity analyses. In mediation analysis, only the functional connectivity between ventral medial prefrontal cortex and right lateral parietal cortex was found as a significant mediator between the CTQ score and HA score (Figure 3B).

### Intra-DMN Functional Connectivity

**Figure 4:**
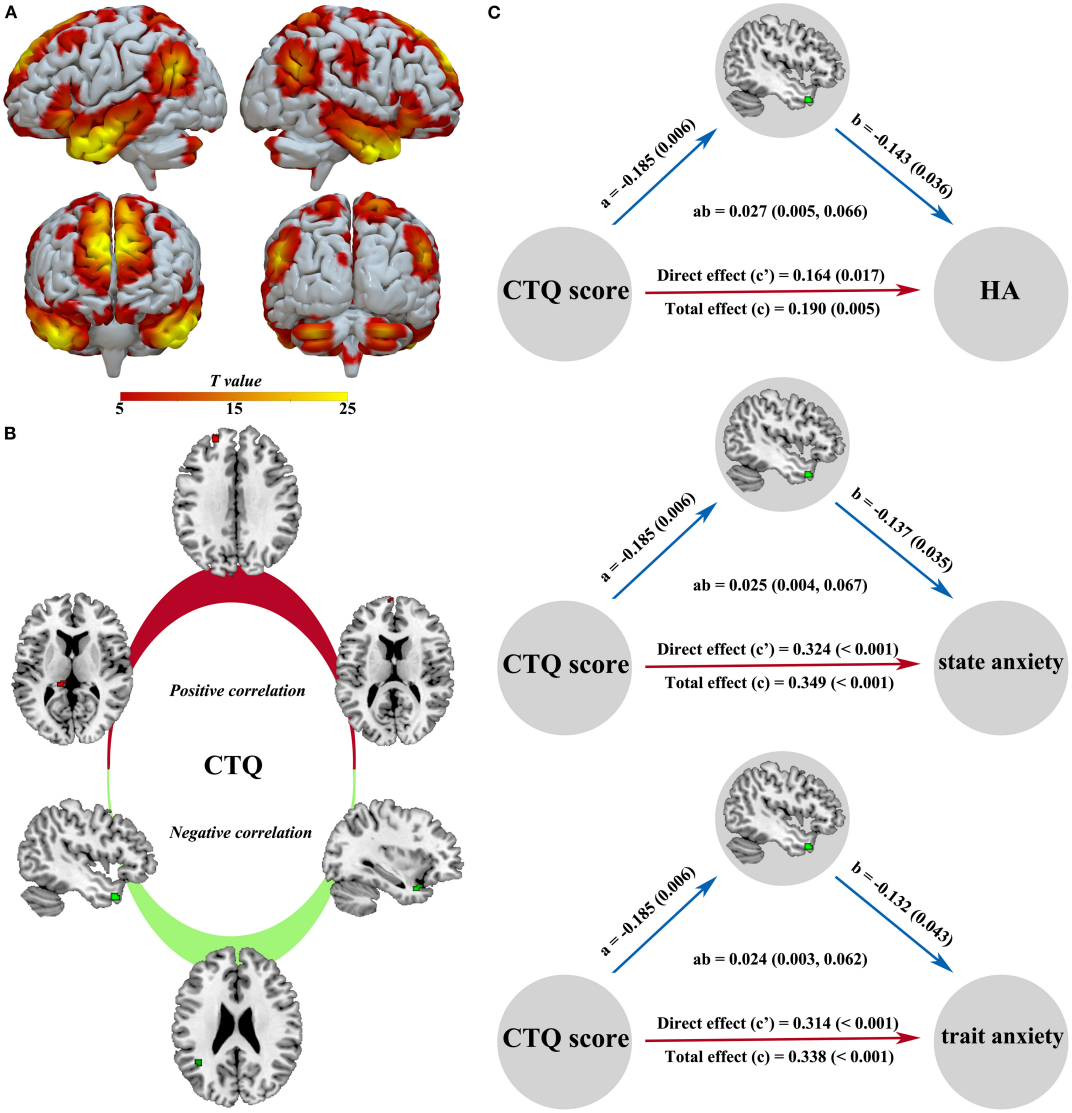
Intra-DMN functional alterations related to the CTQ score and significant mediation effects in the environment-brain-behavior pathway. A sample-specific spatial map of the DMN component is present in **(A)**. The CTQ score was positively related to the left hippocampus-thalamus region, left superior frontal gyrus, and left medial frontal gyrus, while the CTQ score was negatively correlated with the left temporal pole and left angular gyrus **(B)**. The connectivity strength of left temporal pole was found as a significant mediator between the CTQ score and emotional behavior **(C)**. There were significant positive effects from the CTQ score to HA, state anxiety, and trait anxiety, a negative effect from the CTQ score to temporal pole connectivity, and negative effects from the temporal pole connectivity to HA, state anxiety, and trait anxiety. CTQ, Childhood Trauma Questionnaire; DMN, default mode network; HA, harm avoidance.

A sample-specific spatial map of the DMN is present in Figure 4A. The CTQ score was positively related to the left hippocampus-thalamus region (*z* = 2.98, *p* = 0.020), left superior frontal gyrus (*z* = 2.95, *p* = 0.022), and left medial frontal gyrus (*z* = 2.63, *p* = 0.049), while the CTQ score was negatively correlated with the left temporal pole regions (*z* = 2.73, *p* = 0.038; *z* = 2.71, *p* = 0.040) and left angular gyrus (*z* = 2.75, *p* = 0.036) (Figure 4B). In mediation analysis, the connectivity strength of left temporal pole cluster was found as a significant mediator between the CTQ score and behavioral scores (Figure 4C).

### Imaging-Genomic Correlation Analysis

We noted that only the expression level of serotonin transporter gene (SLC6A4) was significantly associated with CTQ-related changes in the DMN component (*R*^2^ = 0.409) (Supplementary Figure S2). The Table 2 summarizes the functional relevance of the SLC6A4 gene based on Metascape gene analysis.

**Table 2:** Functional relevance of the SLC6A4 gene based on GO database, where up to three most informative GO terms were kept, and KEGG pathway.

**Name**	**Description**
Biological process (GO)	GO:0014064 positive regulation of serotonin secretion
	GO:0090067 regulation of thalamus size
	GO:0021941 negative regulation of cerebellar granule cell precursor proliferation
Cellular component (GO)	GO:0099154 serotonergic synapse
	GO:0099056 integral component of presynaptic membrane
	GO:0098889 intrinsic component of presynaptic membrane
Molecular function (GO)	GO:0019811 cocaine binding
	GO:0005335 serotonin:sodium symporter activity
	GO:0008504 monoamine transmembrane transporter activity
KEGG pathway	hsa04721 Synaptic vesicle cycle
	hsa04726 Serotonergic synapse
	hsa05016 Huntington disease

*GO, gene ontology; KEGG, Kyoto Encyclopedia of Genes and Genomes; SLC6A4, serotonin transporter gene*.

## Discussion

Our study is exploratory research using resting-state functional connectivity with a focus on the changes of DMN connectivity related to early trauma. We found dysregulated DMN connectivity both in seed-level and voxel-level functional analyses. Moreover, the functional disruption in the left temporal pole, right parahippocampal gyrus, and frontoparietal connectivity mediated effects of the CTQ score on emotional behavior. The expression of SLC6A4 gene was found associated with DMN alterations induced by childhood trauma, and might suggest the biological underpinning of the relationship between childhood trauma, DMN, and emotion regulation.

### DMN Connectivity Modulated by Childhood Trauma Experience

The DMN intrinsically comprises hubs and distinct subsystems (31). In static functional analysis, functional properties of the anterior medial prefrontal cortex core, inferior temporal sensory subsystem, and self-memory subsystem within the DMN were discriminatively impacted by childhood trauma experience. As a midline core within the DMN, the anterior medial prefrontal cortex exhibits hublike properties in functional analysis (32). It appears to subserve cognitive and affective processes inherent to self-appraisal decisions (33). We found the CTQ score was positively associated with functional pathways of anterior medial prefrontal cortex. As a consequence of hyper-reactivity, the atypical anterior medial prefrontal cortex function may lead to cognitive and affective biases. The inferior temporal cortex is a key part of the ventral visual pathway (34). The CTQ score was negatively associated with the connectivity strength and efficiency of inferior temporal cortex, possibly implying poor processing of adverse sensory in victims. Interestingly, the betweenness centrality in bilateral parahippocampus was differentially impacted by childhood trauma. Dissociated communication and coordination of bilateral parahippocampus may underlie the core pathology in memory-guided disorders. Intentional memory control has considerable implications for mental disorders and may be impaired in victims who suffer from traumatic memories (35). To a certain extent, reduced functional communication of parahippocampus in this study may demonstrate that the capacity to control adverse memory breaks down after experiencing childhood trauma, increasing the tendency of HA personality. The HA personality, a trait associated with behavioral inhibition, plays a key role in the etiology and maintenance of aberrant avoidance behaviors in anxiety (36) and sub-clinical depression (37).

Dynamic analyses provide additional information that is different from the static functional connectivity. In spatial components 1 and 3, the connectivity between bilateral inferior temporal cortex and the superior frontal cortex-parahippocampus connectivity was significantly positively correlated with the CTQ score. The inferior temporal connectivity presented different associations with the CTQ score in static and dynamic analyzes. It may be linked to functional instability of inferior temporal sensory subsystem of DMN. Moreover, the altered dynamic connectivity in the parahippocampus and superior frontal gyrus is consistent with the findings in a previous study (38), supporting the disturbances of inhibitory control and memory encoding in trauma-exposed group. In spatial component 2, the frontoparietal connectivity was significantly negatively correlated with the CTQ score and mediated the relationship between childhood trauma and HA personality. As a neural basis for the ability to create positive meaning from negative experience (39), the ventral medial prefrontal cortex engages in negative affect evaluation and has been shown to be activated robustly by adverse context (40, 41). The lateral parietal lobule, anatomically and functionally connected with ventral medial prefrontal cortex, processes goal-directed attention in human defensive survival circuitry (41). The disrupted frontoparietal connectivity may exaggerate negative emotion, and mediate the development of pessimistic personality in adults with childhood trauma.

We found the intra-DMN connectivity was differently modulated by childhood trauma experience. The hippocampus-thalamus region and prefrontal cortex show positive correlation, which is a part of specific neural networks, and is related to stress response and emotional process (17, 42–44). It may well-indicate that childhood trauma is important for the development of stress sensitization and consequent emotional regulation to daily stress in adult life. The angular gyrus and temporal pole within the DMN showed negative correlation, and are important in visual speech and autobiographical memory (45, 46). Both of them hold important positions in the emotional process of psychiatric patients related to traumatic experience (47–49). Especially, through the mediation effect analysis, reduced connectivity of the temporal pole due to early trauma has deleterious effects on the risk for anxiety and HA personality.

In this community sample, the widespread DMN changes may be explained by functional plasticity enabling adaptation to the early environment, while the environment-brain-behavior pathway mediated by the disrupted connectivity highlights the deleterious effects on emotional behavior.

### Genetic Moderator of Emotion Regulation Related to the DMN Alterations

Both life experience and genes can modify brain development and predict psychopathology. In this study, we further identified that the expression of SLC6A4 gene was associated with the effects of childhood trauma on DMN. The role of SLC6A4 is responsible for serotonin re-uptake, and has received much attention in emotion dysfunction characterized by disrupted serotonergic signaling. Serotonergic neurotransmission modulated by the SLC6A4 is reported regulating impulse control potentially through effects on the DMN (50). A previous study provides further neurochemical evidence that the serotonin system dose-dependently modulates DMN functional connectivity (51). The specific genetic influence of SLC6A4 on anxiety sensitivity has also been well-demonstrated, and this effect is modified by the severity of childhood trauma (52). Combined with gene expression profiles, our research may suggest the serotonergic neuromodulation mechanism, by which the altered DMN connectivity mediates the influence of childhood trauma on behavior.

### Limitations

There are several limitations that should be noted. First, the limitations from the sample itself are mainly shown as heavily weighted toward females, small means of behavioral scores, and small standard deviation of data. It may be that the non-smoking and non-drinking habits in the inclusion criteria reduced the number of male subjects. Although there were no gender differences in behavioral scores and sex was controlled in our statistical model, the effects of sex on psychopathology should be analyzed in future large-sample neuroimaging genetic studies (53). Additionally, since our subjects come from the community, behavioral scores are within the normal or subclinical range, showing small standard deviation and low mean of clinical scales. Our findings offer preliminary evidence that the DMN changes contribute to predicting the tendency of psychopathology in adults with childhood trauma experience. More variable clinical data are needed in the future to verify the associations among traumatic experience, altered DMN connectivity, and psychosis severity. Second, our sample size is relatively small, so we only focus on the imaging-genomic correlations of a large number of candidate risk genes rather than whole-genome, which explores new unknown findings and requires more than thousands of samples to achieve reliable results. It will be important for future research to examine genome-wide differences in gene expression profiles associated with childhood trauma and DMN in a large sample. Next, the gene expression of ABA donors may not completely and accurately reflect the actual gene expression level of subjects. However, considering the certain consistency of gene expression in the process of human evolution, this method of gene expression correlation analysis has still been widely used (54–56). Finally, our imaging-genomic correlation analysis is relatively simple and elementary, and more sophisticated analysis should be considered in the future (57, 58).

## Conclusion

Our findings corroborate the notion that childhood trauma is not only a phenomenon in the early life but has long-term effects on the DMN function and emotional behavior in adulthood. The altered DMN mediated the vulnerability of developing emotion dysfunction in individuals who suffered childhood trauma. The SLC6A4 gene, which regulates emotional responsivity, correlated with the DMN changes and may suggest the related biological mechanism. In summary, the DMN connectivity obtained by different analytical techniques may be useful to provide impressible and psychological relevant information, and can be studied as imaging biomarkers in the pathological processes associated with childhood trauma. Moreover, a clearer understanding of the genetic modulation mechanism of DMN changes, induced by childhood trauma, may be useful in developing pharmacogenomics.

## Data Availability Statement

The datasets used and/or analyzed during the current study are available from the corresponding author on reasonable request.

## Ethics Statement

The studies involving human participants were reviewed and approved by Tongji Hospital, Tongji Medical College, Huazhong University of Science and Technology. The patients/participants provided their written informed consent to participate in this study.

## Author Contributions

TT made substantial contributions to conception and design, acquisition of the data, and analysis and interpretation of the data. JL, GZ, JW, DL, CW, JF, DW, YZ, and YQ made substantial contributions to acquisition of the data, and analysis and interpretation of the data. WZ was involved in drafting the manuscript and revising it critically for important intellectual content. All authors agree to be accountable for the content of the work.

## Funding

This study was supported by National Natural Science Foundation of China (grant numbers: 81601475, 81730049, and 81873890).

## Conflict of Interest

The authors declare that the research was conducted in the absence of any commercial or financial relationships that could be construed as a potential conflict of interest.

## Publisher's Note

All claims expressed in this article are solely those of the authors and do not necessarily represent those of their affiliated organizations, or those of the publisher, the editors and the reviewers. Any product that may be evaluated in this article, or claim that may be made by its manufacturer, is not guaranteed or endorsed by the publisher.
