# Supplementary material for: Default Mode Network Alterations Induced by Childhood Trauma Correlate With Emotional Function and SLC6A4 Expression
Source: Front Psychiatry. 2022 Jan 27;12:760411. doi: 10.3389/fpsyt.2021.760411 (PMC8828908; doi:10.3389/fpsyt.2021.760411)
Supplement: Supplementary file 1 [file Table_1.PDF]

Supplementary Material

1 Supplementary Tables

Table S1. Seed regions for the DMN.

Regions	MNI Coordinates (x, y, z)
amPFC	-3, 54, 18
L.Sup.F	-15, 54, 42
R.Sup.F	18, 42, 48
vmPFC	-6, 36, -9
L.IT	-60, -9, -24
R.IT	57, 0, -27
L.PHC	-24, -18, -27
R.PHC	27, -18, -24
PCC	-3, -48, 30
Rsp	9, -54, 12
L.Lat.P	-48, -69, 39
R.Lat.P	48, -66, 36
Cereb	-6, -54, -48

amPFC: anterior medial prefrontal cortex; Cereb: cerebellar tonsils; DMN: default mode network; L.IT: left inferior temporal cortex; L.Lat.P: left lateral parietal cortex; L.PHC: left parahippocampal gyrus; L.Sup.F: left superior frontal cortex; MNI: Montreal Neurological Institute; PCC: posterior cingulate cortex; R.IT: right inferior temporal cortex; R.Lat.P: right lateral parietal cortex; R.PHC: right parahippocampal gyrus; Rsp: retrosplenial cortex; R.Sup.F: right superior frontal cortex; vmPFC: ventral medial prefrontal cortex.
