# Supplementary material for: Default Mode Network Alterations Induced by Childhood Trauma Correlate With Emotional Function and SLC6A4 Expression
Source: Front Psychiatry. 2022 Jan 27;12:760411. doi: 10.3389/fpsyt.2021.760411 (PMC8828908; doi:10.3389/fpsyt.2021.760411)
Supplement: Supplementary file 2 [file Table_2.PDF]

Supplementary Material

1 Supplementary Tables

Table S2. Mainly psychiatric risk genes associated with childhood trauma within the past ten years.

Risk genes	References	Research type	Psychopathology
NR3C1, FKBP5, SLC6A4, BDNF	(1)	review	Depression
OXTR	(2, 3)	review	Anxiety
MAOA	(4)	meta-analysis	Antisocial behavior
FKBP5, CRHR1, BDNF, OXTR	(5)	review	Various psychiatric disorders
FKBP5, CRHR1, BDNF, MAOA	(6)	review	Mood Disorders
OXTR	(7-9)	original article	Borderline personality disorder, aggression, Suicide ideation
CACNA1C	(10)	original article	Bipolar disorder
NR3C1, DGKH	(11)	original article	Bipolar disorders
FKBP5	(12-25)	original article	Depression, dissociative symptoms, suicide,<br>post-traumatic stress disorder, aggressive behavior
CCL3, CRP, CSF2, IFNG, IL12B, IL1A, IL4, IL6, TNF	(26)	original article	Panic disorder

IL1B	(27-29)	original article	Depression, internalizing symptoms
CRHR1, FKBP5, NR3C1, NR3C2	(30)	original article	Depression
NR3C1, TNF, IL1B	(31)	original article	Major depressive disorder
CRHR1	(32)	original article	Depression
MAOA, 5-HTT	(33)	original article	Aggression tendencies
MAOA	(34-42)	original article	Psychopathy, aggressive behavior, personality pathology, polysubstance use
NR3C1	(43-45)	original article	Borderline personality disorder, major depressive disorder, post-traumatic stress disorder
CRHBP, FKBP5	(46)	original article	Suicidal behavior in bipolar patients
5-HTT/ SLC6A4	(47-62)	original article	Depression,<br><br>antisocial personality disorder, callous and unemotional traits, suicidal behavior, anxiety, alexithymia, empathic perspective, stress
5-HTT, CRHR1, BDNF, CREB1, FKBP5, IL1B, NTRK2, OXTR	(63)	review	Depression
COMT	(64-71)	original article	Suicidal behaviour, cannabis use, schizotypal personality, startle reflex , panic, anxiety, anger
CRHR1	(72-74)	original article	Neuroticism, depression, emotional empathy

FKBP5, CRH, NR3C2	(75)	original article	Stress-related disorders
OXTR, DRD4	(76)	original article	Depression
MAOA, BDNF, COMT	(77)	original article	Hyperactivity symptoms
BDNF	(78-85)	original article	Major depressive disorder, suicide attempt, anxiety, schizophrenia, bipolar disorder, obsessive-compulsive disorder, stress
RGS2	(86)	original article	Anxiety and depressive disorders
CRP, IL1b, IL6, IL11, TNF	(87)	original article	Depression
IL6, IL-1β, NF-Kb	(88)	original article	Stress
GABRA2	(89)	original article	Drug dependence
DAT1, MAOA, ADRB2, DBH, SLC6A2, 5HTR3A, COMT, 5-HTT, TPH1, BDNF, FKBP5, OXTR, NR3C1, CRHR	(90)	review	Borderline personality disorder
NR3C1, BDNF, OXTR	(91)	original article	Schizophrenia
NR3C1	(92, 93)	original article	Major depressive disorder, anxiety, substance-use disorders.
NR3C2, NR3C1	(94)	original article	Cocaine addiction
NPSR1	(95)	original	Anxiety

		article	
FKBP5, CRHR1, NR3C1	(96)	original article	Stress
5-HT3A R	(97)	original article	Bipolar, borderline personality, attention deficit hyperactivity disorders
MAOA, COMT	(98)	original article	Non-suicidal self-injury
CRHBP, FKBP5	(99)	original article	Suicide
FKBP5, OXTR	(100)	original article	Borderline personality
5-HTT, BDNF	(101-106)	original article	Depression, suicide
GABBR1, GRIN2D, CACNA2D4, PSEN2	(107)	original article	Various psychiatric disorders
CREB1	(108)	original article	Anger traits
TPH2, 5-HTT	(109)	original article	Depression
RORA	(110)	original article	Anxiety
VEGFA, TOLLIP, SIRT1	(111)	original article	Depression
COMT, MAOA, MAOB	(112)	original article	Obsessive-compulsive disorder
TLR2	(113)	original article	Bipolar disorders
NR3C1, FKBP5	(114)	original	Borderline personality disorder

		article	
5-HTT, MAOA	(115)	original article	Psychopathic traits
DRD2	(116)	original article	Bulimia-spectrum disorder
TPH1, 5-HTT, MAOA	(117)	original article	Antisocial behavior
DISC1	(118)	original article	Schizophrenia
CRHR1, CRHR2	(119)	original article	Suicide

References

1. Park, C., Rosenblat, J. D., Brietzke, E., Pan, Z., Lee, Y., Cao, B., et al. (2019). Stress, epigenetics and depression: A systematic review. Neurosci Biobehav Rev. 102: 139-152. doi:10.1016/j.neubiorev.2019.04.010

2. Gottschalk, M. G., Domschke, K. (2018). Oxytocin and Anxiety Disorders. Curr Top Behav Neurosci. 35: 467-498. doi:10.1007/7854_2017_25

3. Womersley, J. S., Hemmings, S. M. J., Ziegler, C., Gutridge, A., Ahmed-Leitao, F., Rosenstein, D., et al. (2020). Childhood emotional neglect and oxytocin receptor variants: Association with limbic brain volumes. World J Biol Psychiatry. 21(7): 513-528. doi:10.1080/15622975.2019.1584331

4. Byrd, A. L., Manuck, S. B. (2014). MAOA, childhood maltreatment, and antisocial behavior: meta-analysis of a gene-environment interaction. Biol Psychiatry. 75(1): 9-17. doi:10.1016/j.biopsych.2013.05.004

5. Nemeroff, C. B. (2016). Paradise Lost: The Neurobiological and Clinical Consequences of Child Abuse and Neglect. Neuron. 89(5): 892-909. doi:10.1016/j.neuron.2016.01.019

6. Lippard, E. T. C., Nemeroff, C. B. (2020). The Devastating Clinical Consequences of Child Abuse and Neglect: Increased Disease Vulnerability and Poor Treatment Response in Mood Disorders. Am J Psychiatry. 177(1): 20-36. doi:10.1176/appi.ajp.2019.19010020

7. Zhang, M., Liu, N., Chen, H., Zhang, N. (2020). Oxytocin receptor gene, childhood maltreatment and borderline personality disorder features among male inmates in China. BMC Psychiatry. 20(1): 332. doi:10.1186/s12888-020-02710-0

8. Zhang, Y., Wu, C., Chang, H., Yan, Q., Wu, L., Yuan, S., et al. (2018). Genetic variants in oxytocin receptor gene (OXTR) and childhood physical abuse collaborate to modify the risk of aggression in chinese adolescents. J Affect Disord. 229: 105-110. doi:10.1016/j.jad.2017.12.024

9. Handley, E. D., Warmingham, J. M., Rogosch, F. A., Cicchetti, D. (2019). Infancy onset maltreatment and the development of suicide ideation: An investigation of moderation by oxytocin-related gene polymorphisms. J Affect Disord. 257: 421-427. doi:10.1016/j.jad.2019.06.051

10. Bastos, C. R., Tovo-Rodrigues, L., Ardais, A. P., Xavier, J., Salerno, P. S. V., Camerini, L., et al. (2020). The role of CACNA1C gene and childhood trauma interaction on bipolar disorder. Prog Neuropsychopharmacol Biol Psychiatry. 101: 109915. doi:10.1016/j.pnpbp.2020.109915

11. Grillault Laroche, D., Curis, E., Bellivier, F., Nepost, C., Courtin, C., Etain, B., et al. (2020). Childhood maltreatment and HPA axis gene expression in bipolar disorders: A gene network analysis. Psychoneuroendocrinology. 120: 104753. doi:10.1016/j.psyneuen.2020.104753

12. Klinger-Konig, J., Hertel, J., Van der Auwera, S., Frenzel, S., Pfeiffer, L., Waldenberger, M., et al. (2019). Methylation of the FKBP5 gene in association with FKBP5 genotypes, childhood maltreatment and depression. Neuropsychopharmacology. 44(5): 930-938. doi:10.1038/s41386-019-0319-6

13. Tozzi, L., Carballedo, A., Wetterling, F., McCarthy, H., O'Keane, V., Gill, M., et al. (2016). Single-Nucleotide Polymorphism of the FKBP5 Gene and Childhood Maltreatment as Predictors of Structural Changes in Brain Areas Involved in Emotional Processing in Depression. Neuropsychopharmacology. 41(2): 487-97. doi:10.1038/npp.2015.170

14. Yaylaci, F. T., Cicchetti, D., Rogosch, F. A., Bulut, O., Hetzel, S. R. (2017). The interactive effects of child maltreatment and the FK506 binding protein 5 gene (FKBP5) on dissociative symptoms in adolescence. Dev Psychopathol. 29(3): 1105-1117. doi:10.1017/S095457941600105X

15. Roy, A., Gorodetsky, E., Yuan, Q., Goldman, D., Enoch, M. A. (2010). Interaction of FKBP5, a stress-related gene, with childhood trauma increases the risk for attempting suicide. Neuropsychopharmacology. 35(8): 1674-83. doi:10.1038/npp.2009.236

16. Kang, C., Shi, J., Gong, Y., Wei, J., Zhang, M., Ding, H., et al. (2020). Interaction between FKBP5 polymorphisms and childhood trauma on depressive symptoms in Chinese adolescents: The moderating role of resilience. J Affect Disord. 266: 143-150. doi:10.1016/j.jad.2020.01.051

17. Tozzi, L., Farrell, C., Booij, L., Doolin, K., Nemoda, Z., Szyf, M., et al. (2018). Epigenetic Changes of FKBP5 as a Link Connecting Genetic and Environmental Risk Factors with Structural and Functional Brain Changes in Major Depression. Neuropsychopharmacology. 43(5): 1138-1145. doi:10.1038/npp.2017.290

18. Kohrt, B. A., Worthman, C. M., Ressler, K. J., Mercer, K. B., Upadhaya, N., Koirala, S., et al. (2015). Cross-cultural gene- environment interactions in depression, post-traumatic stress disorder, and the cortisol awakening response: FKBP5 polymorphisms and childhood trauma in South Asia. Int Rev Psychiatry. 27(3): 180-96. doi:10.3109/09540261.2015.1020052

19. Appel, K., Schwahn, C., Mahler, J., Schulz, A., Spitzer, C., Fenske, K., et al. (2011). Moderation of adult depression by a polymorphism in the FKBP5 gene and childhood physical abuse in the general population. Neuropsychopharmacology. 36(10): 1982-91. doi:10.1038/npp.2011.81

20. Mihaljevic, M., Franic, D., Soldatovic, I., Lukic, I., Petrovic, S. A., Mirjanic, T., et al. (2021). The FKBP5 genotype and childhood trauma effects on FKBP5 DNA methylation in patients with psychosis, their unaffected siblings, and healthy controls. Psychoneuroendocrinology. 128: 105205. doi:10.1016/j.psyneuen.2021.105205

21. Xie, P., Kranzler, H. R., Poling, J., Stein, M. B., Anton, R. F., Farrer, L. A., et al. (2010). Interaction of FKBP5 with childhood adversity on risk for post-traumatic stress disorder. Neuropsychopharmacology. 35(8): 1684-92. doi:10.1038/npp.2010.37

22. Handley, E. D., Rogosch, F. A., Cicchetti, D. (2015). Developmental pathways from child maltreatment to adolescent marijuana dependence: Examining moderation by FK506 binding protein 5 gene (FKBP5). Dev Psychopathol. 27(4 Pt 2): 1489-502. doi:10.1017/S0954579415000899

23. Bevilacqua, L., Carli, V., Sarchiapone, M., George, D. K., Goldman, D., Roy, A., et al. (2012). Interaction between FKBP5 and childhood trauma and risk of aggressive behavior. Arch Gen Psychiatry. 69(1): 62-70. doi:10.1001/archgenpsychiatry.2011.152

24. Dackis, M. N., Rogosch, F. A., Oshri, A., Cicchetti, D. (2012). The role of limbic system irritability in linking history of childhood maltreatment and psychiatric outcomes in low-income, high-risk women: moderation by FK506 binding protein 5 haplotype. Dev Psychopathol. 24(4): 1237-52. doi:10.1017/S0954579412000673

25. Misiak, B., Karpinski, P., Szmida, E., Grazlewski, T., Jablonski, M., Cyranka, K., et al. (2020). Adverse Childhood Experiences and Methylation of the FKBP5 Gene in Patients with Psychotic Disorders. J Clin Med. 9(12). doi:10.3390/jcm9123792

26. Zou, Z., Huang, Y., Wang, J., Min, W., Zhou, B. (2020). DNA methylation of IL-4 gene and the association with childhood trauma in panic disorder. Psychiatry Res. 293: 113385. doi:10.1016/j.psychres.2020.113385

27. Chen, Y., Zhang, Z., Xu, Z., Pu, M., Geng, L. (2015). [Influence of interleukin-1 beta gene polymorphism and childhood maltreatment on antidepressant treatment]. Zhonghua Yi Xue Yi Chuan Xue Za Zhi. 32(6): 801-4. doi:10.3760/cma.j.issn.1003-9406.2015.06.010

28. McQuaid, R. J., Gabrys, R. L., McInnis, O. A., Anisman, H., Matheson, K. (2019). Understanding the Relation Between Early-Life Adversity and Depression Symptoms: The Moderating Role of Sex and an Interleukin-1beta Gene Variant. Front Psychiatry. 10: 151. doi:10.3389/fpsyt.2019.00151

29. Ridout, K. K., Parade, S. H., Seifer, R., Price, L. H., Gelernter, J., Feliz, P., et al. (2014). Interleukin 1B gene (IL1B) variation and internalizing symptoms in maltreated preschoolers. Dev Psychopathol. 26(4 Pt 2): 1277-87. doi:10.1017/S0954579414001023

30. Normann, C., Buttenschon, H. N. (2020). Gene-environment interactions between HPA-axis genes and childhood maltreatment in depression: a systematic review. Acta Neuropsychiatr: 1-11. doi:10.1017/neu.2020.1

31. Spindola, L. M., Pan, P. M., Moretti, P. N., Ota, V. K., Santoro, M. L., Cogo-Moreira, H., et al. (2017). Gene expression in blood of children and adolescents: Mediation between childhood maltreatment and major depressive disorder. J Psychiatr Res. 92: 24-30. doi:10.1016/j.jpsychires.2017.03.015

32. Grabe, H. J., Schwahn, C., Appel, K., Mahler, J., Schulz, A., Spitzer, C., et al. (2010). Childhood maltreatment, the corticotropin-releasing hormone receptor gene and adult depression in the general population. Am J Med Genet B Neuropsychiatr Genet. 153B(8): 1483-93. doi:10.1002/ajmg.b.31131

33. Zhang, Y., Ming, Q. S., Yi, J. Y., Wang, X., Chai, Q. L., Yao, S. Q. (2017). Gene-Gene-Environment Interactions of Serotonin Transporter, Monoamine Oxidase A and Childhood

Maltreatment Predict Aggressive Behavior in Chinese Adolescents. Front Behav Neurosci. 11: 17. doi:10.3389/fnbeh.2017.00017

34. Hollerbach, P., Johansson, A., Ventus, D., Jern, P., Neumann, C. S., Westberg, L., et al. (2018). Main and interaction effects of childhood trauma and the MAOA uVNTR polymorphism on psychopathy. Psychoneuroendocrinology. 95: 106-112. doi:10.1016/j.psyneuen.2018.05.022

35. Checknita, D., Bendre, M., Ekstrom, T. J., Comasco, E., Tiihonen, J., Hodgins, S., et al. (2020). Monoamine oxidase A genotype and methylation moderate the association of maltreatment and aggressive behaviour. Behav Brain Res. 382: 112476. doi:10.1016/j.bbr.2020.112476

36. Haberstick, B. C., Lessem, J. M., Hewitt, J. K., Smolen, A., Hopfer, C. J., Halpern, C. T., et al. (2014). MAOA genotype, childhood maltreatment, and their interaction in the etiology of adult antisocial behaviors. Biol Psychiatry. 75(1): 25-30. doi:10.1016/j.biopsych.2013.03.028

37. Byrd, A. L., Manuck, S. B., Hawes, S. W., Vebares, T. J., Nimgaonkar, V., Chowdari, K. V., et al. (2018). The interaction between monoamine oxidase A (MAOA) and childhood maltreatment as a predictor of personality pathology in females: Emotional reactivity as a potential mediating mechanism. Dev Psychopathol: 1-17. doi:10.1017/S0954579417001900

38. Fite, P. J., Brown, S., Hossain, W. A., Manzardo, A., Butler, M. G., Bortolato, M. (2019). Sex-Dimorphic Interactions of MAOA Genotype and Child Maltreatment Predispose College Students to Polysubstance Use. Front Genet. 10: 1314. doi:10.3389/fgene.2019.01314

39. Fite, P. J., Brown, S., Hossain, W., Manzardo, A., Butler, M. G., Bortolato, M. (2019). Tobacco and cannabis use in college students are predicted by sex-dimorphic interactions between MAOA genotype and child abuse. CNS Neurosci Ther. 25(1): 101-111. doi:10.1111/cns.13002

40. Fergusson, D. M., Boden, J. M., Horwood, L. J., Miller, A. L., Kennedy, M. A. (2011). MAOA, abuse exposure and antisocial behaviour: 30-year longitudinal study. Br J Psychiatry. 198(6): 457-63. doi:10.1192/bjp.bp.110.086991

41. Zhang, Y., Ming, Q., Wang, X., Yao, S. (2016). The interactive effect of the MAOA-VNTR genotype and childhood abuse on aggressive behaviors in Chinese male adolescents. Psychiatr Genet. 26(3): 117-23. doi:10.1097/YPG.0000000000000125

42. Gorodetsky, E., Bevilacqua, L., Carli, V., Sarchiapone, M., Roy, A., Goldman, D., et al. (2014). The interactive effect of MAOA-LPR genotype and childhood physical neglect on aggressive behaviors in Italian male prisoners. Genes Brain Behav. 13(6): 543-9. doi:10.1111/gbb.12140

43. Perroud, N., Paoloni-Giacobino, A., Prada, P., Olie, E., Salzmann, A., Nicastro, R., et al. (2011). Increased methylation of glucocorticoid receptor gene (NR3C1) in adults with a history of childhood maltreatment: a link with the severity and type of trauma. Transl Psychiatry. 1: e59. doi:10.1038/tp.2011.60

44. Martin-Blanco, A., Ferrer, M., Soler, J., Salazar, J., Vega, D., Andion, O., et al. (2014). Association between methylation of the glucocorticoid receptor gene, childhood maltreatment, and clinical severity in borderline personality disorder. J Psychiatr Res. 57: 34-40. doi:10.1016/j.jpsychires.2014.06.011

45. Perroud, N., Dayer, A., Piguet, C., Nallet, A., Favre, S., Malafosse, A., et al. (2014). Childhood maltreatment and methylation of the glucocorticoid receptor gene NR3C1 in bipolar disorder. Br J Psychiatry. 204(1): 30-5. doi:10.1192/bjp.bp.112.120055

46. Segura, A. G., Mitjans, M., Jimenez, E., Fatjo-Vilas, M., Ruiz, V., Saiz, P. A., et al. (2019). Association of childhood trauma and genetic variability of CRH-BP and FKBP5 genes with suicidal behavior in bipolar patients. J Affect Disord. 255: 15-22. doi:10.1016/j.jad.2019.05.014

47. Brown, G. W., Ban, M., Craig, T. K., Harris, T. O., Herbert, J., Uher, R. (2013). Serotonin transporter length polymorphism, childhood maltreatment, and chronic depression: a specific gene-environment interaction. Depress Anxiety. 30(1): 5-13. doi:10.1002/da.21982

48. Onoue, T., Toda, H., Nakai, Y. (2013). [Childhood stress and depression]. Nihon Shinkei Seishin Yakurigaku Zasshi. 33(3): 105-10.

49. Uher, R., Caspi, A., Houts, R., Sugden, K., Williams, B., Poulton, R., et al. (2011). Serotonin transporter gene moderates childhood maltreatment's effects on persistent but not single-episode depression: replications and implications for resolving inconsistent results. J Affect Disord. 135(1-3): 56-65. doi:10.1016/j.jad.2011.03.010

50. Cutuli, J. J., Raby, K. L., Cicchetti, D., Englund, M. M., Egeland, B. (2013). Contributions of maltreatment and serotonin transporter genotype to depression in childhood, adolescence, and early adulthood. J Affect Disord. 149(1-3): 30-7. doi:10.1016/j.jad.2012.08.011

51. Antypa, N., Van der Does, A. J. (2010). Serotonin transporter gene, childhood emotional abuse and cognitive vulnerability to depression. Genes Brain Behav. 9(6): 615-20. doi:10.1111/j.1601-183X.2010.00593.x

52. Grabe, H. J., Schwahn, C., Mahler, J., Schulz, A., Spitzer, C., Fenske, K., et al. (2012). Moderation of adult depression by the serotonin transporter promoter variant (5-HTTLPR), childhood abuse and adult traumatic events in a general population sample. Am J Med Genet B Neuropsychiatr Genet. 159B(3): 298-309. doi:10.1002/ajmg.b.32027

53. Banducci, A. N., Gomes, M., MacPherson, L., Lejuez, C. W., Potenza, M. N., Gelernter, J., et al. (2014). A Preliminary Examination of the Relationship Between the 5-HTTLPR and Childhood Emotional Abuse on Depressive Symptoms in 10-12-Year-Old Youth. Psychol Trauma. 6(1): 1-7. doi:10.1037/a0031121

54. Douglas, K., Chan, G., Gelernter, J., Arias, A. J., Anton, R. F., Poling, J., et al. (2011). 5-HTTLPR as a potential moderator of the effects of adverse childhood experiences on risk of antisocial personality disorder. Psychiatr Genet. 21(5): 240-8. doi:10.1097/YPG.0b013e3283457c15

55. Widom, C. S., Miller, D., Li, X., Gordon, D., Brzustowicz, L. (2020). Childhood maltreatment, serotonin transporter gene, and risk for callous and unemotional traits: A prospective investigation. Psychiatry Res. 291: 113271. doi:10.1016/j.psychres.2020.113271

56. Roy, A., Hu, X. Z., Janal, M. N., Goldman, D. (2007). Interaction between childhood trauma and serotonin transporter gene variation in suicide. Neuropsychopharmacology. 32(9): 2046-52. doi:10.1038/sj.npp.1301331

57. Enoch, M. A., Hodgkinson, C. A., Gorodetsky, E., Goldman, D., Roy, A. (2013). Independent effects of 5' and 3' functional variants in the serotonin transporter gene on suicidal behavior in the context of childhood trauma. J Psychiatr Res. 47(7): 900-7. doi:10.1016/j.jpsychires.2013.03.007

58. Schiele, M. A., Ziegler, C., Holitschke, K., Schartner, C., Schmidt, B., Weber, H., et al. (2016). Influence of 5-HTT variation, childhood trauma and self-efficacy on anxiety traits: a gene-environment-coping interaction study. J Neural Transm (Vienna). 123(8): 895-904. doi:10.1007/s00702-016-1564-z

59. Klauke, B., Deckert, J., Reif, A., Pauli, P., Zwanzger, P., Baumann, C., et al. (2011). Serotonin transporter gene and childhood trauma--a G x E effect on anxiety sensitivity. Depress Anxiety. 28(12): 1048-57. doi:10.1002/da.20840

60. Terock, J., Weihs, A., Teumer, A., Klinger-Konig, J., Janowitz, D., Grabe, H. J. (2021). Associations and interactions of the serotonin receptor genes 5-HT1A, 5-HT2A, and childhood trauma with alexithymia in two independent general-population samples. Psychiatry Res. 298: 113783. doi:10.1016/j.psychres.2021.113783

61. Flasbeck, V., Moser, D., Pakusch, J., Kumsta, R., Brune, M. (2019). The association between childhood maltreatment and empathic perspective taking is moderated by the 5-HTT linked polymorphic region: Another example of "differential susceptibility". PLoS One. 14(12): e0226737. doi:10.1371/journal.pone.0226737

62. Harkness, K. L., Bagby, R. M., Stewart, J. G., Larocque, C. L., Mazurka, R., Strauss, J. S., et al. (2015). Childhood emotional and sexual maltreatment moderate the relation of the serotonin transporter gene to stress generation. J Abnorm Psychol. 124(2): 275-87. doi:10.1037/abn0000034

63. Li, M., Liu, S., D'Arcy, C., Gao, T., Meng, X. (2020). Interactions of childhood maltreatment and genetic variations in adult depression: A systematic review. J Affect Disord. 276: 119-136. doi:10.1016/j.jad.2020.06.055

64. Kwon, A., Min, D., Kim, Y., Jin, M. J., Lee, S. H. (2020). Interaction between catechol-O-methyltransferase polymorphism and childhood trauma in suicidal ideation of patients with post-traumatic stress disorder. Brain Behav. 10(8): e01733. doi:10.1002/brb3.1733

65. Bernegger, A., Kienesberger, K., Carlberg, L., Swoboda, P., Ludwig, B., Koller, R., et al. (2018). The Impact of COMT and Childhood Maltreatment on Suicidal Behaviour in Affective Disorders. Sci Rep. 8(1): 692. doi:10.1038/s41598-017-19040-z

66. Vinkers, C. H., Van Gastel, W. A., Schubart, C. D., Van Eijk, K. R., Luykx, J. J., Van Winkel, R., et al. (2013). The effect of childhood maltreatment and cannabis use on adult psychotic symptoms is modified by the COMT Val(1)(5)(8)Met polymorphism. Schizophr Res. 150(1): 303-11. doi:10.1016/j.schres.2013.07.020

67. Savitz, J., van der Merwe, L., Newman, T. K., Stein, D. J., Ramesar, R. (2010). Catechol-o-methyltransferase genotype and childhood trauma may interact to impact schizotypal personality traits. Behav Genet. 40(3): 415-23. doi:10.1007/s10519-009-9323-7

68. Klauke, B., Winter, B., Gajewska, A., Zwanzger, P., Reif, A., Herrmann, M. J., et al. (2012). Affect-modulated startle: interactive influence of catechol-O-methyltransferase Val158Met genotype and childhood trauma. PLoS One. 7(6): e39709. doi:10.1371/journal.pone.0039709

69. Asselmann, E., Hertel, J., Beesdo-Baum, K., Schmidt, C. O., Homuth, G., Nauck, M., et al. (2018). Interplay between COMT Val158Met, childhood adversities and sex in predicting panic pathology: Findings from a general population sample. J Affect Disord. 234: 290-296. doi:10.1016/j.jad.2018.02.060

70. Baumann, C., Klauke, B., Weber, H., Domschke, K., Zwanzger, P., Pauli, P., et al. (2013). The interaction of early life experiences with COMT val158met affects anxiety sensitivity. Genes Brain Behav. 12(8): 821-9. doi:10.1111/gbb.12090

71. Perroud, N., Jaussent, I., Guillaume, S., Bellivier, F., Baud, P., Jollant, F., et al. (2010). COMT but not serotonin-related genes modulates the influence of childhood abuse on anger traits. Genes Brain Behav. 9(2): 193-202. doi:10.1111/j.1601-183X.2009.00547.x

72. DeYoung, C. G., Cicchetti, D., Rogosch, F. A. (2011). Moderation of the association between childhood maltreatment and neuroticism by the corticotropin-releasing hormone receptor 1 gene. J Child Psychol Psychiatry. 52(8): 898-906. doi:10.1111/j.1469-7610.2011.02404.x

73. Laucht, M., Treutlein, J., Blomeyer, D., Buchmann, A. F., Schmidt, M. H., Esser, G., et al. (2013). Interactive effects of corticotropin-releasing hormone receptor 1 gene and childhood adversity on depressive symptoms in young adults: findings from a longitudinal study. Eur Neuropsychopharmacol. 23(5): 358-67. doi:10.1016/j.euroneuro.2012.06.002

74. Grimm, S., Wirth, K., Fan, Y., Weigand, A., Gartner, M., Feeser, M., et al. (2017). The interaction of corticotropin-releasing hormone receptor gene and early life stress on emotional empathy. Behav Brain Res. 329: 180-185. doi:10.1016/j.bbr.2017.04.047

75. Gerritsen, L., Milaneschi, Y., Vinkers, C. H., van Hemert, A. M., van Velzen, L., Schmaal, L., et al. (2017). HPA Axis Genes, and Their Interaction with Childhood Maltreatment, are Related to Cortisol Levels and Stress-Related Phenotypes. Neuropsychopharmacology. 42(12): 2446-2455. doi:10.1038/npp.2017.118

76. Cao, H., Zhou, N., Leerkes, E. M., Su, J. (2021). The etiology of maternal postpartum depressive symptoms: Childhood emotional maltreatment, couple relationship satisfaction, and genes. J Fam Psychol. 35(1): 44-56. doi:10.1037/fam0000722

77. Tsai, M. C., Jhang, K. J., Lee, C. T., Lin, Y. F., Strong, C., Lin, Y. C., et al. (2020). Effects of Childhood Adversity and Its Interaction with the MAOA, BDNF, and COMT Polymorphisms on Subclinical Attention Deficit/Hyperactivity Symptoms in Generally Healthy Youth. Children (Basel). 7(9). doi:10.3390/children7090122

78. Ferrer, A., Labad, J., Salvat-Pujol, N., Barrachina, M., Costas, J., Urretavizcaya, M., et al. (2019). BDNF genetic variants and methylation: effects on cognition in major depressive disorder. Transl Psychiatry. 9(1): 265. doi:10.1038/s41398-019-0601-8

79. Perroud, N., Courtet, P., Vincze, I., Jaussent, I., Jollant, F., Bellivier, F., et al. (2008). Interaction between BDNF Val66Met and childhood trauma on adult's violent suicide attempt. Genes Brain Behav. 7(3): 314-22. doi:10.1111/j.1601-183X.2007.00354.x

80. Min, J. A., Lee, H. J., Lee, S. H., Park, Y. M., Kang, S. G., Chae, J. H. (2013). Gender-specific effects of brain-derived neurotrophic factor Val66Met polymorphism and childhood maltreatment on anxiety. Neuropsychobiology. 67(1): 6-13. doi:10.1159/000342384

81. Aas, M., Haukvik, U. K., Djurovic, S., Tesli, M., Athanasiu, L., Bjella, T., et al. (2014). Interplay between childhood trauma and BDNF val66met variants on blood BDNF mRNA levels and on hippocampus subfields volumes in schizophrenia spectrum and bipolar disorders. J Psychiatr Res. 59: 14-21. doi:10.1016/j.jpsychires.2014.08.011

82. Hemmings, S. M., Lochner, C., van der Merwe, L., Cath, D. C., Seedat, S., Stein, D. J. (2013). BDNF Val66Met modifies the risk of childhood trauma on obsessive-compulsive disorder. J Psychiatr Res. 47(12): 1857-63. doi:10.1016/j.jpsychires.2013.08.012

83. Perea, C. S., Paternina, A. C., Gomez, Y., Lattig, M. C. (2012). Negative affectivity moderated by BDNF and stress response. J Affect Disord. 136(3): 767-74. doi:10.1016/j.jad.2011.09.043

84. Carballedo, A., Morris, D., Zill, P., Fahey, C., Reinhold, E., Meisenzahl, E., et al. (2013). Brain-derived neurotrophic factor Val66Met polymorphism and early life adversity affect hippocampal volume. Am J Med Genet B Neuropsychiatr Genet. 162B(2): 183-90. doi:10.1002/ajmg.b.32130

85. Miller, S., Hallmayer, J., Wang, P. W., Hill, S. J., Johnson, S. L., Ketter, T. A. (2013). Brain-derived neurotrophic factor val66met genotype and early life stress effects upon bipolar course. J Psychiatr Res. 47(2): 252-8. doi:10.1016/j.jpsychires.2012.10.015

86. Asselmann, E., Hertel, J., Schmidt, C. O., Homuth, G., Nauck, M., Beesdo-Baum, K., et al. (2018). Interplay between RGS2 and childhood adversities in predicting anxiety and depressive disorders: Findings from a general population sample. Depress Anxiety. 35(11): 1104-1113. doi:10.1002/da.22812

87. Cohen-Woods, S., Fisher, H. L., Ahmetspahic, D., Douroudis, K., Stacey, D., Hosang, G. M., et al. (2018). Interaction between childhood maltreatment on immunogenetic risk in depression: Discovery and replication in clinical case-control samples. Brain Behav Immun. 67: 203-210. doi:10.1016/j.bbi.2017.08.023

88. Schreier, H. M. C., Kuras, Y. I., McInnis, C. M., Thoma, M. V., St Pierre, D. G., Hanlin, L., et al. (2020). Childhood Physical Neglect Is Associated With Exaggerated Systemic and Intracellular Inflammatory Responses to Repeated Psychosocial Stress in Adulthood. Front Psychiatry. 11: 504. doi:10.3389/fpsyt.2020.00504

89. Enoch, M. A., Hodgkinson, C. A., Yuan, Q., Shen, P. H., Goldman, D., Roy, A. (2010). The influence of GABRA2, childhood trauma, and their interaction on alcohol, heroin, and cocaine dependence. Biol Psychiatry. 67(1): 20-7. doi:10.1016/j.biopsych.2009.08.019

90. Wilson, N., Robb, E., Gajwani, R., Minnis, H. (2021). Nature and nurture? A review of the literature on childhood maltreatment and genetic factors in the pathogenesis of borderline personality disorder. J Psychiatr Res. 137: 131-146. doi:10.1016/j.jpsychires.2020.12.025

91. Barker, V., Walker, R. M., Evans, K. L., Lawrie, S. M. (2020). Methylation of glucocorticoid receptor (NR3C1), BDNF and oxytocin receptor genes in association with childhood maltreatment in schizophrenia and schizoaffective disorder. Schizophr Res. 216: 529-531. doi:10.1016/j.schres.2019.11.050

92. Bustamante, A. C., Aiello, A. E., Galea, S., Ratanatharathorn, A., Noronha, C., Wildman, D. E., et al. (2016). Glucocorticoid receptor DNA methylation, childhood maltreatment and major depression. J Affect Disord. 206: 181-188. doi:10.1016/j.jad.2016.07.038

93. Tyrka, A. R., Parade, S. H., Welch, E. S., Ridout, K. K., Price, L. H., Marsit, C., et al. (2016). Methylation of the leukocyte glucocorticoid receptor gene promoter in adults: associations with early adversity and depressive, anxiety and substance-use disorders. Transl Psychiatry. 6(7): e848. doi:10.1038/tp.2016.112

94. Rovaris, D. L., Mota, N. R., Bertuzzi, G. P., Aroche, A. P., Callegari-Jacques, S. M., Guimaraes, L. S., et al. (2015). Corticosteroid receptor genes and childhood neglect influence susceptibility to crack/cocaine addiction and response to detoxification treatment. J Psychiatr Res. 68: 83-90. doi:10.1016/j.jpsychires.2015.06.008

95. Schiele, M. A., Herzog, K., Kollert, L., Schartner, C., Leehr, E. J., Bohnlein, J., et al. (2020). Extending the vulnerability-stress model of mental disorders: three-dimensional NPSR1 x

environment x coping interaction study in anxiety. Br J Psychiatry. 217(5): 645-650. doi:10.1192/bjp.2020.73

96. Ramo-Fernandez, L., Boeck, C., Koenig, A. M., Schury, K., Binder, E. B., Gundel, H., et al. (2019). The effects of childhood maltreatment on epigenetic regulation of stress-response associated genes: an intergenerational approach. Sci Rep. 9(1): 983. doi:10.1038/s41598-018-36689-2

97. Perroud, N., Zewdie, S., Stenz, L., Adouan, W., Bavamian, S., Prada, P., et al. (2016). Methylation of Serotonin Receptor 3a in Adhd, Borderline Personality, and Bipolar Disorders: Link with Severity of the Disorders and Childhood Maltreatment. Depress Anxiety. 33(1): 45-55. doi:10.1002/da.22406

98. Gao, Y., Xiong, Y., Liu, X., Wang, H. (2021). The Effects of Childhood Maltreatment on Non-Suicidal Self-Injury in Male Adolescents: The Moderating Roles of the Monoamine Oxidase A (MAOA) Gene and the Catechol-O-Methyltransferase (COMT) Gene. Int J Environ Res Public Health. 18(5). doi:10.3390/ijerph18052598

99. Roy, A., Hodgkinson, C. A., Deluca, V., Goldman, D., Enoch, M. A. (2012). Two HPA axis genes, CRHBP and FKBP5, interact with childhood trauma to increase the risk for suicidal behavior. J Psychiatr Res. 46(1): 72-9. doi:10.1016/j.jpsychires.2011.09.009

100. Cicchetti, D., Rogosch, F. A., Hecht, K. F., Crick, N. R., Hetzel, S. (2014). Moderation of maltreatment effects on childhood borderline personality symptoms by gender and oxytocin receptor and FK506 binding protein 5 genes. Dev Psychopathol. 26(3): 831-49. doi:10.1017/S095457941400042X

101. Gutierrez, B., Bellon, J. A., Rivera, M., Molina, E., King, M., Marston, L., et al. (2015). The risk for major depression conferred by childhood maltreatment is multiplied by BDNF and SERT genetic vulnerability: a replication study. J Psychiatry Neurosci. 40(3): 187-96. doi:10.1503/jpn.140097

102. Benedetti, F., Ambree, O., Locatelli, C., Lorenzi, C., Poletti, S., Colombo, C., et al. (2017). The effect of childhood trauma on serum BDNF in bipolar depression is modulated by the serotonin promoter genotype. Neurosci Lett. 656: 177-181. doi:10.1016/j.neulet.2017.07.043

103. Harkness, K. L., Strauss, J., Michael Bagby, R., Stewart, J. G., Larocque, C., Mazurka, R., et al. (2015). Interactions between childhood maltreatment and brain-derived neurotrophic factor and serotonin transporter polymorphisms on depression symptoms. Psychiatry Res. 229(1-2): 609-12. doi:10.1016/j.psychres.2015.04.040

104. Jimenez-Trevino, L., Saiz, P. A., Garcia-Portilla, M. P., Blasco-Fontecilla, H., Carli, V., Iosue, M., et al. (2019). 5-HTTLPR-brain-derived neurotrophic factor (BDNF) gene interactions and early adverse life events effect on impulsivity in suicide attempters. World J Biol Psychiatry. 20(2): 137-149. doi:10.1080/15622975.2017.1376112

105. Grabe, H. J., Schwahn, C., Mahler, J., Appel, K., Schulz, A., Spitzer, C., et al. (2012). Genetic epistasis between the brain-derived neurotrophic factor Val66Met polymorphism and the 5-HTT promoter polymorphism moderates the susceptibility to depressive disorders after childhood abuse. Prog Neuropsychopharmacol Biol Psychiatry. 36(2): 264-70. doi:10.1016/j.pnpbp.2011.09.010

106. Comasco, E., Aslund, C., Oreland, L., Nilsson, K. W. (2013). Three-way interaction effect of 5-HTTLPR, BDNF Val66Met, and childhood adversity on depression: a replication study. Eur Neuropsychopharmacol. 23(10): 1300-6. doi:10.1016/j.euroneuro.2013.01.010

107. Cecil, C. A., Smith, R. G., Walton, E., Mill, J., McCrory, E. J., Viding, E. (2016). Epigenetic signatures of childhood abuse and neglect: Implications for psychiatric vulnerability. J Psychiatr Res. 83: 184-194. doi:10.1016/j.jpsychires.2016.09.010

108. Hasler, R., Perroud, N., Baud, P., Olie, E., Guillaume, S., Malafosse, A., et al. (2012). CREB1 modulates the influence of childhood sexual abuse on adult's anger traits. Genes Brain Behav. 11(6): 720-6. doi:10.1111/j.1601-183X.2012.00807.x

109. Van der Auwera, S., Janowitz, D., Schulz, A., Homuth, G., Nauck, M., Volzke, H., et al. (2014). Interaction among childhood trauma and functional polymorphisms in the serotonin pathway moderate the risk of depressive disorders. Eur Arch Psychiatry Clin Neurosci. 264 Suppl 1: S45-54. doi:10.1007/s00406-014-0536-2

110. Min, J. A., Lee, H. J., Lee, S. H., Park, Y. M., Kang, S. G., Park, Y. G., et al. (2017). RORA Polymorphism Interacts with Childhood Maltreatment in Determining Anxiety Sensitivity by Sex: A Preliminary Study in Healthy Young Adults. Clin Psychopharmacol Neurosci. 15(4): 402-406. doi:10.9758/cpn.2017.15.4.402

111. Lo Iacono, L., Bussone, S., Andolina, D., Tambelli, R., Troisi, A., Carola, V. (2020). Dissecting major depression: The role of blood biomarkers and adverse childhood experiences in distinguishing clinical subgroups. J Affect Disord. 276: 351-360. doi:10.1016/j.jad.2020.07.034

112. McGregor, N. W., Hemmings, S. M. J., Erdman, L., Calmarza-Font, I., Stein, D. J., Lochner, C. (2016). Modification of the association between early adversity and obsessive-compulsive disorder by polymorphisms in the MAOA, MAOB and COMT genes. Psychiatry Res. 246: 527-532. doi:10.1016/j.psychres.2016.10.044

113. Oliveira, J., Etain, B., Lajnef, M., Hamdani, N., Bennabi, M., Bengoufa, D., et al. (2015). Combined effect of TLR2 gene polymorphism and early life stress on the age at onset of bipolar disorders. PLoS One. 10(3): e0119702. doi:10.1371/journal.pone.0119702

114. Flasbeck, V., Brune, M. (2021). Association between childhood maltreatment, psychopathology and DNA methylation of genes involved in stress regulation: Evidence from a study in Borderline Personality Disorder. PLoS One. 16(3): e0248514. doi:10.1371/journal.pone.0248514

115. Sadeh, N., Javdani, S., Verona, E. (2013). Analysis of monoaminergic genes, childhood abuse, and dimensions of psychopathy. J Abnorm Psychol. 122(1): 167-79. doi:10.1037/a0029866

116. Groleau, P., Joober, R., Israel, M., Zeramdini, N., DeGuzman, R., Steiger, H. (2014). Methylation of the dopamine D2 receptor (DRD2) gene promoter in women with a bulimia-spectrum disorder: associations with borderline personality disorder and exposure to childhood abuse. J Psychiatr Res. 48(1): 121-7. doi:10.1016/j.jpsychires.2013.10.003

117. Cicchetti, D., Rogosch, F. A., Thibodeau, E. L. (2012). The effects of child maltreatment on early signs of antisocial behavior: genetic moderation by tryptophan hydroxylase, serotonin transporter, and monoamine oxidase A genes. Dev Psychopathol. 24(3): 907-28. doi:10.1017/S0954579412000442

118. Sayin, A., Yuksel, N., Konac, E., Yilmaz, A., Dogan, B., Tonge, S., et al. (2013). Effects of the adverse life events and Disrupted in Schizophrenia-1 (DISC1) gene polymorphisms on acute symptoms of schizophrenia. DNA Cell Biol. 32(2): 73-80. doi:10.1089/dna.2012.1894

119. Guillaume, S., Perroud, N., Jollant, F., Jaussent, I., Olie, E., Malafosse, A., et al. (2013). HPA axis genes may modulate the effect of childhood adversities on decision-making in suicide attempters. J Psychiatr Res. 47(2): 259-65. doi:10.1016/j.jpsychires.2012.10.014
