# Supplementary material for: Default Mode Network Alterations Induced by Childhood Trauma Correlate With Emotional Function and SLC6A4 Expression
Source: Front Psychiatry. 2022 Jan 27;12:760411. doi: 10.3389/fpsyt.2021.760411 (PMC8828908; doi:10.3389/fpsyt.2021.760411)
Supplement: Supplementary file 3 [file Image_1.PDF]

Supplementary Material

1.1 Supplementary Figures

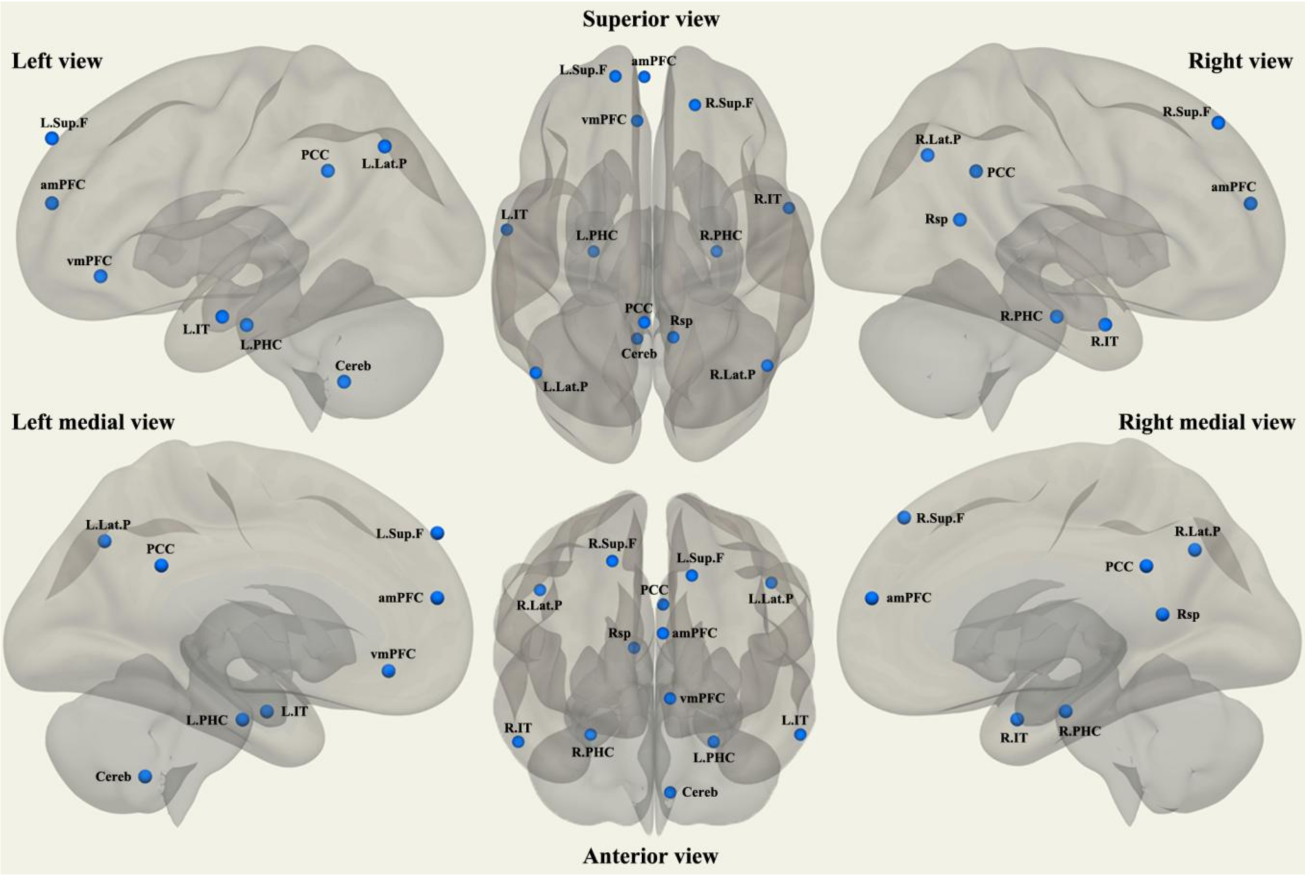

Supplementary Figure 1. Distribution of each seed within the DMN on a three-dimensional map. amPFC: anterior medial prefrontal cortex; Cereb: cerebellar tonsils; DMN: default mode network; L.IT: left inferior temporal cortex; L.Lat.P: left lateral parietal cortex; L.PHC: left parahippocampal gyrus; L.Sup.F: left superior frontal cortex; PCC: posterior cingulate cortex; R.IT: right inferior temporal cortex; R.Lat.P: right lateral parietal cortex; R.PHC: right parahippocampal gyrus; Rsp: retrosplenial cortex; R.Sup.F: right superior frontal cortex; vmPFC: ventral medial prefrontal cortex.
