# Supplementary material for: Default Mode Network Alterations Induced by Childhood Trauma Correlate With Emotional Function and SLC6A4 Expression
Source: Front Psychiatry. 2022 Jan 27;12:760411. doi: 10.3389/fpsyt.2021.760411 (PMC8828908; doi:10.3389/fpsyt.2021.760411)
Supplement: Supplementary file 4 [file Image_2.pdf]

Supplementary Material

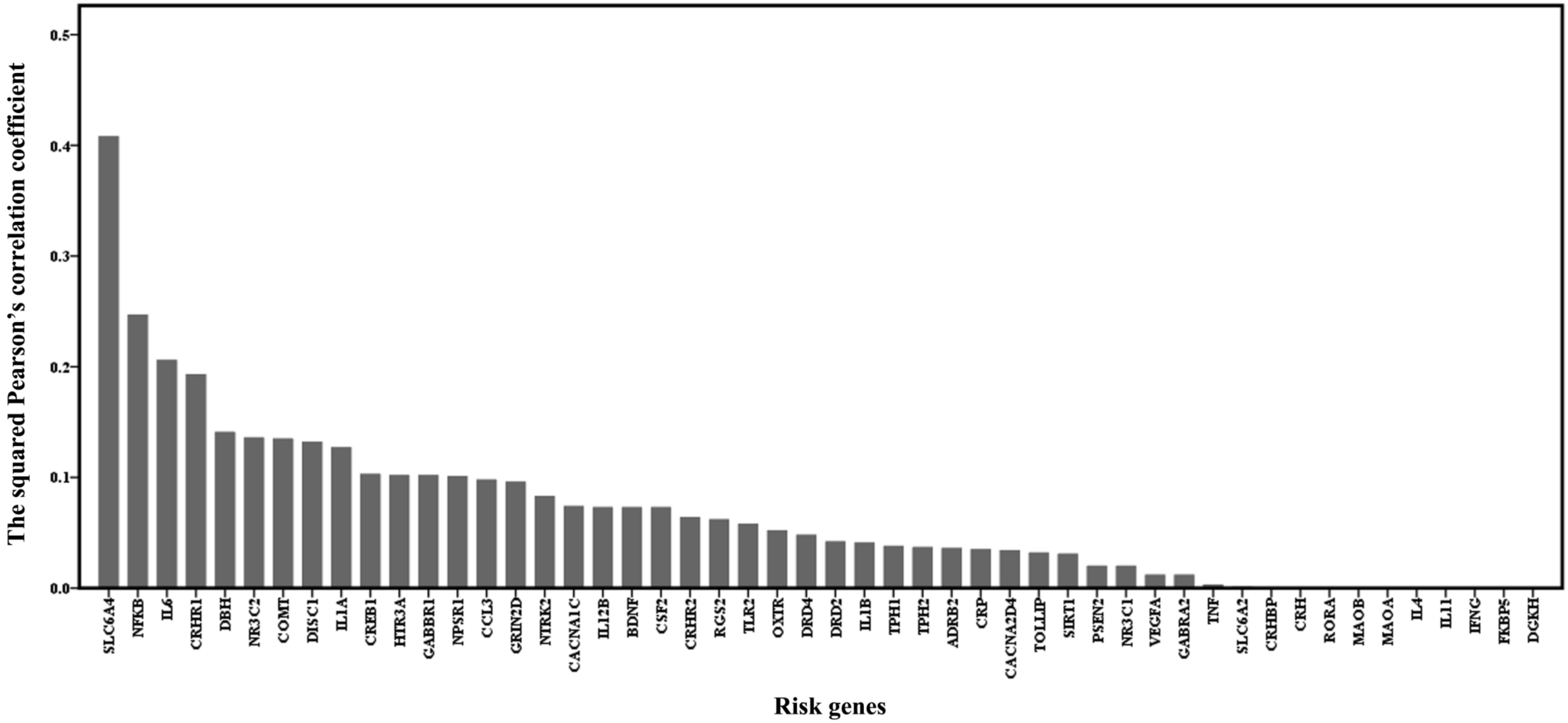

Supplementary Figure 2. Correlations between the expression level for each risk gene and statistic mapping derived from regression analysis. The expression of SLC6A4 gene was significantly associated with CTQ-related changes in the DMN component. CTQ:~~-~~; Childhood Trauma Questionnaire; DMN: default mode network; ~~SLC6A4~~serotonin transporter gene.

SLC6A4:
